# Supplementary material for: Novel Driver Strength Index highlights important cancer genes in TCGA PanCanAtlas patients
Source: PeerJ. 2022 Aug 11;10:e13860. doi: 10.7717/peerj.13860 (PMC9375969; doi:10.7717/peerj.13860)
Supplement: Supplemental Information 2 [file peerj-10-13860-s002.zip › COHORTS/genes plots/age/2021_8_16_14_9_distribution_cohorts_females_50-54.pdf]

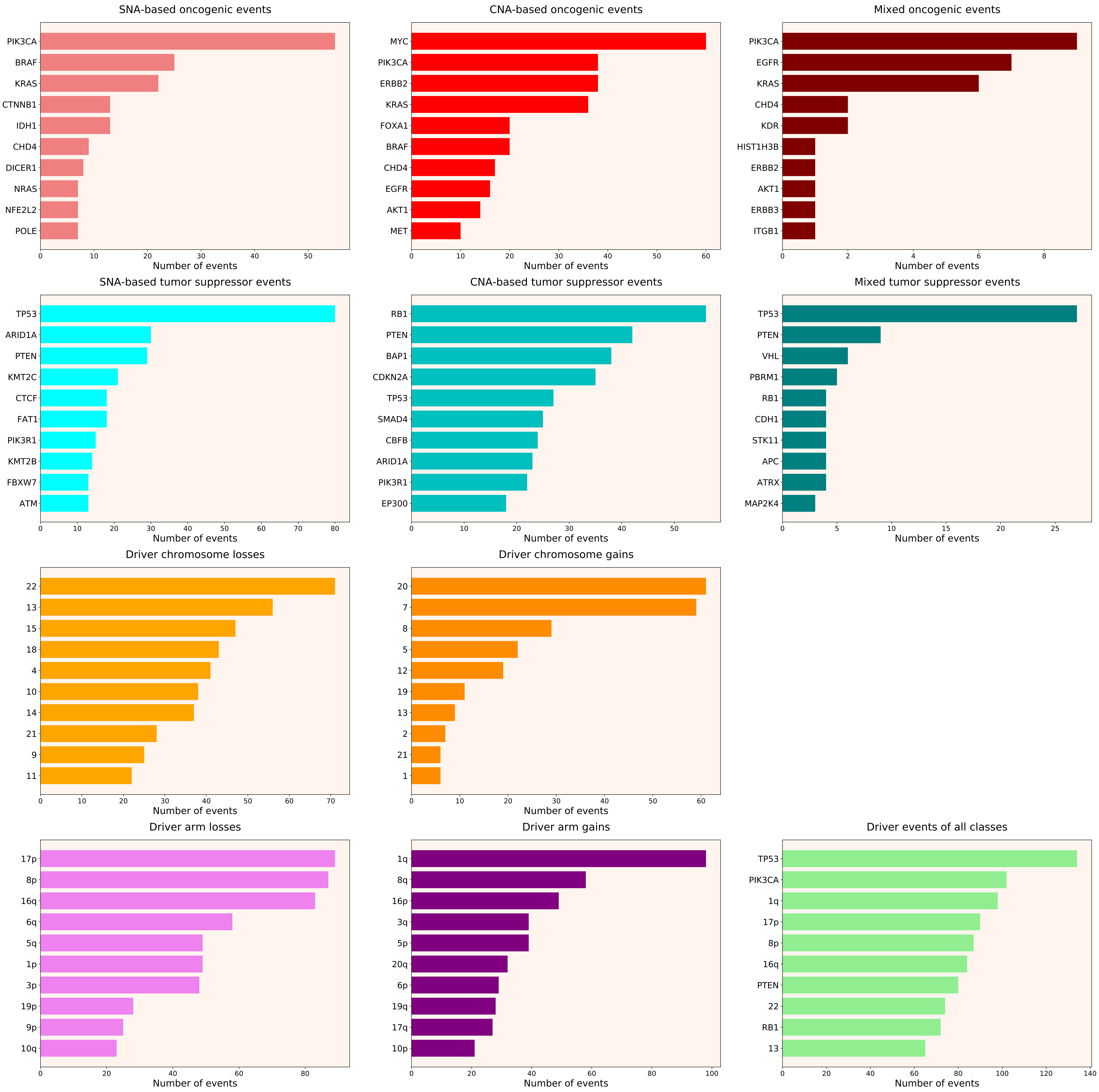

SNA-based oncogenic events
PIK3CA
BRAF
KRAS
CTNNB1
IDH1
CHD4
DICER1
NRAS
NFE2L2
POLE
Number of events
CNA-based oncogenic events
MYC
PIK3CA
ERBB2
KRAS
FOXA1
BRAF
CHD4
EGFR
AKT1
MET
Number of events
Mixed oncogenic events
PIK3CA
EGFR
KRAS
CHD4
KDR
HIST1H3B
ERBB2
AKT1
ERBB3
ITGB1
Number of events
SNA-based tumor suppressor events
TP53
ARID1A
PTEN
KMT2C
CTCF
FAT1
PIK3R1
KMT2B
FBXW7
ATM
Number of events
CNA-based tumor suppressor events
RB1
PTEN
BAP1
CDKN2A
TP53
SMAD4
CBFB
ARID1A
PIK3R1
EP300
Number of events
Mixed tumor suppressor events
TP53
PTEN
VHL
PBRM1
RB1
CDH1
STK11
APC
ATRX
MAP2K4
Number of events
Driver chromosome losses
22
13
15
18
4
10
14
21
9
11
Number of events
Driver chromosome gains
20
7
8
5
12
19
13
2
21
1
Number of events
Driver arm losses
17p
8p
16q
6q
5q
1p
3p
19p
9p
10q
Number of events
Driver arm gains
1q
8q
16p
3q
5p
20q
6p
19q
17q
10p
Number of events
Driver events of all classes
TP53
PIK3CA
1q
17p
8p
16q
PTEN
22
RB1
13
Number of events
